# Development and Validation of Prognostic Nomogram in Patients With WHO Grade III Meningioma: A Retrospective Cohort Study Based on SEER Database

**DOI:** 10.3389/fonc.2021.719974

**Published:** 2021-12-01

**Authors:** Zetian Jia, Yaqi Yan, Jiuxin Wang, He Yang, Haihua Zhan, Qian Chen, Yawei He, Yuhua Hu

**Affiliations:** ^1^ Department of Neurosurgery, The Second Hospital of Hebei Medical University, Shijiazhuang, China; ^2^ Department of Cardiology, The First Hospital of Handan of Hebei Province, Handan, China

**Keywords:** meningioma, surveillance epidemiology and end results, nomogram, prognosis, risk factor

## Abstract

**Introduction:**

World Health Organization (WHO) Grade III meningioma is a central nervous system tumor with a poor prognosis. In this retrospective cohort study, the authors constructed a nomogram for predicting the prognosis of WHO Grade III meningioma.

**Methods:**

The patients of this nomogram were based on the Surveillance, Epidemiology, and End Results (SEER) database between 2000 and 2018. All patients were randomly divided into a development cohort (964 patients) and a validation cohort (410 patients) in a 7:3 ratio. The least absolute shrinkage and selection operator (LASSO) regression was used to screen the predictors. The Cox hazards regression model was constructed and the prognosis was visualized by nomogram. The performance of the prognostic nomogram was determined by consistency index (C-index), clinical net benefit, and calibration.

**Results:**

Eight variables were included in the nomogram: gender, race, age at diagnosis, histology, tumor site, tumor size, laterality, and surgical method. The C-index of the training set and verification set were 0.654 and 0.628. The calibration plots showed that the nomogram was in good agreement with the actual observation. The clinical decision curve indicates that the nomogram has a good clinical net benefit in WHO Grade III meningioma.

**Conclusions:**

A prognostic nomogram of a large cohort of WHO Grade III meningioma was established and verified based on the SEER database. The nomogram we established may help clinicians provide personalized treatment services and clinical decisions for patients.

## Introduction

Meningiomas are the most common primary brain tumors in adults, accounting for 36.4% of central nervous system tumors according to previous reports (1). Although most meningiomas are benign, some high-grade meningiomas have aggressive biological characteristics and a poor prognosis. According to the 2016 edition of the World Health Organization (WHO) Central nervous system classification criteria, WHO Grade III meningioma include papillary meningioma, rhabdoid meningioma, and anaplastic meningioma (2). WHO Grade III meningioma is characterized by the high degree of malignancy, high recurrence rate, and poor clinical prognosis. Evidence from previous studies suggests that the age of patients with meningioma was proposed as an independent prognostic factor (3). Although WHO Grade III meningioma accounts for about 2% of all meningiomas, their poor clinical prognosis has attracted the attention of neuroscientists (4). Some studies have shown that the average survival time of WHO Grade III meningioma is about 3 years (4, 5). It is very necessary to accurately predict the survival time of WHO Grade III meningioma patients, but there is no effective prediction tool. Therefore, an effective and accurate prediction model needs to be developed to help clinicians provide individualized treatment for patients with meningioma.

The Surveillance, Epidemiology and End Results (SEER) database incorporates clinical data from cancer patients from 18 United States (US) registries, representing approximately 28% of the US population. Compared with other single-center studies with small sample sizes, the clinical data from the SEER dataset has a large sample size and complete data, which increases the credibility of the study.

The nomogram can show the constructed proportional hazards regression model in a visual form. The influence coefficient of each prognostic factor was scored, and then the total scores were added to calculate the risk probability of the outcome event. Therefore, Nomogram has high clinical practical value and has been widely used in medical research and clinical practice (6). Considering that there is currently no effective predictive tool for WHO Grade III meningioma patients, in order to better evaluate the influence of risk factors on the prognosis of patients with WHO Grade III meningioma patients, and then provide appropriate treatment strategies for them. We developed and validated a nomogram for the prognosis of WHO Grade III meningioma patients based on a large SEER population database.

## Materials and Methods

### Study Population: SEER Data

The study was based on a population-based retrospective cohort study; Clinical data of the patients were obtained from the SEER database. SEER database is the largest database of cancer in the United States, covering about 28% of the total population. A population data set containing 18 registries was extracted from a subset of the SEER dataset [Nov 2020 Sub (2000–2018)]. Statistical data of clinical patients were obtained by using SEER* Stat 8.3.9 software (username: 10901-Nov2020). International Classification of Diseases for Oncology, third edition (ICD-O-3) behavior codes are not consistent with WHO classification of meningiomas (7). Our definition of WHO Grade III meningioma is based on the central nervous system tumor classification published by the World Health Organization in 2016 (2).

Inclusion criteria are as follows: (1) ICD-O-3 coded: 9530/3 (Meningioma, malignant) and 9538/3 (Papillary meningioma); (2) The diagnosis was confirmed by microscopic examination: “Positive histology” and “Positive microscopic confirm, method not specified”; (3) The primary site of meningioma is coded as: “C70.0-Cerebral meninges”, “C70.1-Spinal meninges”, “ C70.9-Meninges, NOS” and “C71.0-C72.9”; Exclusion criteria: (1) The surgical procedure is unknown: “RX Summ–Surg Prim Site (1998+)” with code 99; (2) Follow-up time was unknown or zero days.

### Study Design

The study was designed based on transparent reporting of a multivariable prediction model for individual prognosis or diagnosis (TRIPOD) (8). The outcome of this study was 3- and 5-year survival status of WHO Grade III meningioma patients. The following clinical data were extracted as potential variables for the prognostic model: gender, age at diagnosis, race, histology type, primary tumor site, tumor size, laterality, surgery modality, follow-up time, and survival status. The age classification criteria are children (0-19 years old), adults (20-59 years old), and elderly (over 60 years old). In this study, racial categories were divided into white, black, and others (American Indian/Alaska Native and Asian/Pacific Islander). Histology categories for this study included malignant meningioma (9530/3) and papillary meningioma (9538/3). The primary site of the tumor was classified as cerebral meninges (C70.0), spinal meninges (C70.1), Meninges not otherwise specified (C70.9), and others (C71.0-C72.9). Tumor sizes were divided into three categories: 0-3.9 cm, 4cm+ and unknown. Tumor laterality was classified as only one side (left-origin of primary; only one side-side unspecified; right-origin of primary) and bilateral side (bilateral, single primary; not a paired site; paired site, but no information concerning laterality; paired site: midline tumor). The surgical methods of this study were classified as no surgery/biopsy only (codes 00, 10, 20), subtotal resection (STR, codes 21, 22, 40, 90), and gross total resection (GTR, codes 30, 55).

Based on the advantage of the large sample size of the SEER database, the development cohort of our prognostic model had a sample size of 964 people and the validation cohort had a sample size of 410 people. Ensure the stability of the model being constructed. If the sample size of the missing value in the database is less than 5% of the total number of people, it will be deleted. If the number of missing values is greater than 5%, the missing values are filled by multiple interpolations using the “mice” package in R software.

### Statistical Analysis

All statistical analyses were performed using R software 4.0.5 (https://www.r-project.org). Patients with WHO Grade III meningioma were randomly divided into a development group and a validation group according to the ratio of 7:3 by “caret” package. Among prognostic risk factors for WHO Grade III meningioma, the least absolute shrinkage and selection operator (LASSO) regression were used to select the best predictor variables for 3- and 5-year survival rates in the development group (9). All the included variables must satisfy the proportional hazards assumption before further constructing the Cox proportional hazards model. Nomogram was used to predict 3- and 5-year survival in WHO Grade III meningioma. The differentiation of clinical prediction models was evaluated by concordance index (C-index). The “rms” package was used to draw calibration curves to evaluate the consistency of the nomogram. The calibration plots were constructed with 1000 repeated samples by bootstrap method. Decision curve analysis (DCA) was used to evaluate the net clinical benefit of the prediction model for WHO Grade III meningioma (10). Cox proportional hazards regression model was established based on the variables included in the model to calculate the 3- and 5-year death probability of WHO Grade III meningioma. The receiver operating characteristic curve (ROC) was constructed with mortality as a continuous variable and the area under the curve (AUC) was calculated. The Kaplan-Meier (KM) survival curve was drawn by dividing the mortality rates into high-risk and low-risk groups using the maximum Jordan index as the best cut-off value. Log-rank tests were used to compare differences between high-risk and low-risk groups. All test results with P values less than 0.05 were considered statistically significant.

## Results

### Patient Baseline Characteristics

**Figure 1:**
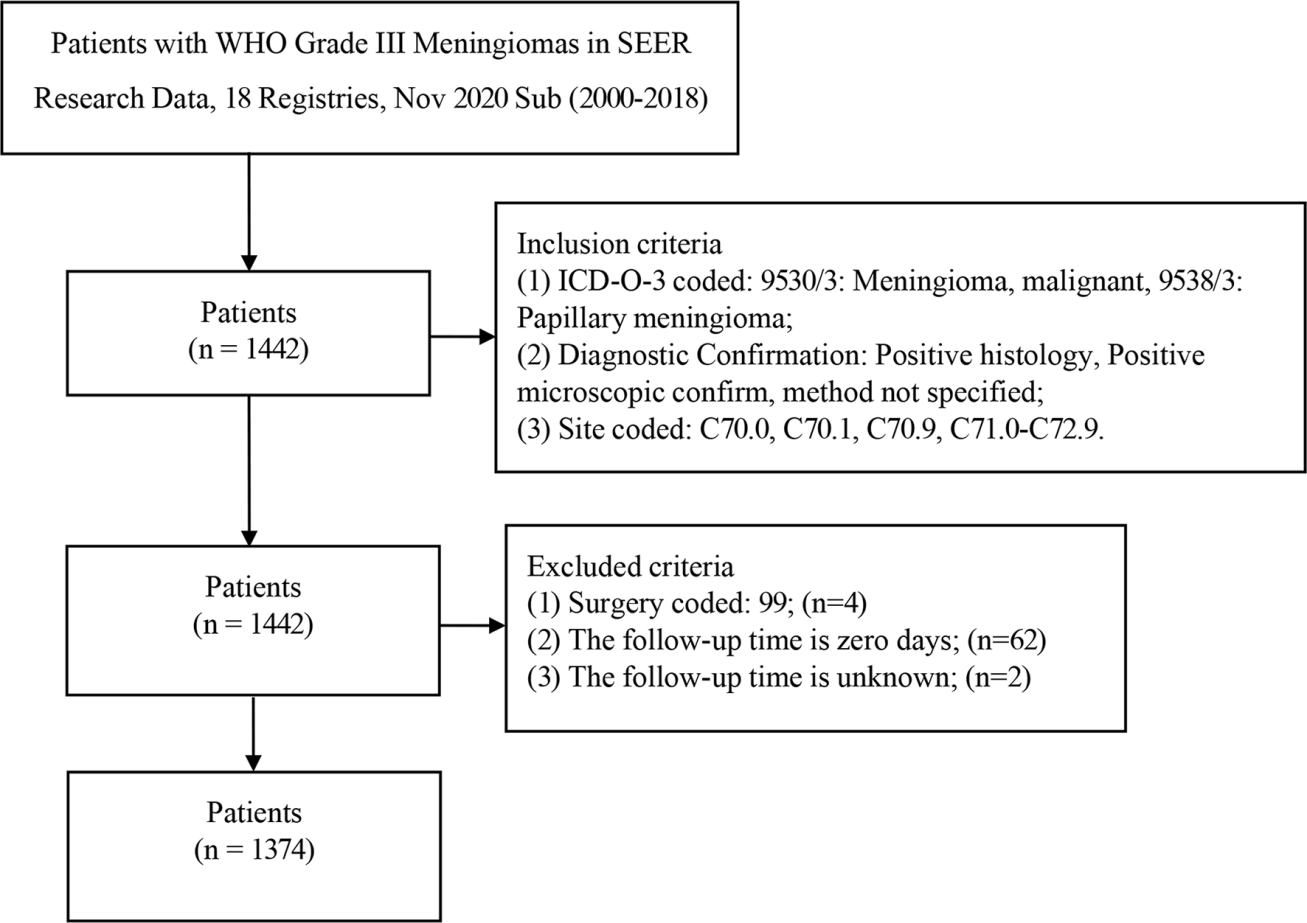
Flowchart of participant inclusion and exclusion.

A total of 1442 patients were diagnosed with WHO Grade III meningioma from 2000 to 2018 in the SEER database. 1374 patients were included in the analysis (development group n=964, validation group n=410; **Table 1**). The number of patients deleted due to incomplete data accounted for about 4.72% of the total patients. The number of males in the development set was 453 (46.99%) and women 511 (53.01%), while the number of men in the validation set was 182 (44.39%) and 228 (55.61%). Previous studies have suggested that age is an influential factor for the prognosis of patients with meningioma (11), so we divided the age into three levels according to 0-19 years old, 20-59 years old, and 60+ years old. Training set according to the number of the age stratification in 15 (1.56%), 418 (43.36%), 531 (55.08%), validation set: 11 (2.68%), 170 (58.0), 229 (40.8%). Race in the development cohort: white 713 (73.96%), black 144 (14.94%) and other 107 (11.10%); Validation cohort: white 298 (72.68%), black 61 (14.88%) and others 51 (12.44%). The number of 9530/3 (Meningioma, malignant) in the development and validation groups was 873 (90.56%) and 368 (89.76), respectively. The number of 9538/3 (Papillary meningioma) in the development set and validation set is 91 (9.44%) and 42 (10.24%). The primary sites of tumors in the training set were classified as follows: Cerebral meninges 765 (79.36%), Spinal meninges 35 (3.63%), Meninges not otherwise specified 124 (12.86%) and others 40 (4.15%); Validation set: Cerebral meninges 327 (79.76%), Spinal meninges 10 (2.44%), Meninges not otherwise specified 54 (14.15%) and others 15 (3.66%); Results of tumor size classification in the development group: 0-3.9 cm 143 (14.83%), 4+ cm 258 (26.76%) and unknown 563(58.40%); Validation group: 0-3.9 cm 62 (15.12%), 4+ cm 122 (29.76%) and unknown 226 (55.12%). The number of one side and bilateral in development cohorts was 602 (62.45%) and 362 (37.55%); The number of one side and bilateral in validation cohorts was 257 (62.68%) and 153 (37.32%). The surgical methods of the training group: No surgery/Biopsy only 270 (28.01%), subtotal resection 278 (28.84%) and gross total resection 416 (43.15%); The surgical methods of the validation group: No surgery/Biopsy only 108 (26.34%), subtotal resection 107 (26.10%) and gross total resection 195 (47.56%). There was no statistical difference between development cohort and validation cohort (P>0.05). Screening details and demographic characteristics are shown in **Figure 1** and **Table 1**, respectively.

**Table 1:** Baseline characteristics of WHO Grade III meningioma patients from SEER database.

Characteristics	All patients N = 1374 (%)	Training set N = 964 (%)	Validation set N = 410 (%)	P value
Follow-up (Months)				0.441
Median (IQR)	48 (16 to 112)	49 (16 to 113)	45 (15 to 110)	
Gender				0.409
Male	635 (46.22)	453 (46.99)	182 (44.39)	
Female	739 (53.78)	511 (53.01)	228 (55.61)	
Age (years)				0.332
0-19	26 (1.89)	15 (1.56)	11 (2.68)	
20-59	588 (42.79)	418 (43.36)	170 (41.46)	
60+	760 (55.31)	531 (55.08)	229 (55.85)	
Race				0.774
White	1011 (73.58)	713 (73.96)	298 (72.68)	
Black	205 (14.92)	144 (14.94)	61 (14.88)	
Others	158 (11.50)	107 (11.10)	51 (12.44)	
Histology				0.718
Meningioma, malignant	1241 (90.32)	873 (90.56)	368 (89.76)	
Papillary meningioma	133 (9.68)	91 (9.44)	42 (10.24)	
Location				0.619
Cerebral meninges	1092 (79.48)	765 (79.36)	327 (79.76)	
Spinal meninges	45 (3.28)	35 (3.63)	10 (2.44)	
Meninges, NOS	182 (13.25)	124 (12.86)	58 (14.15)	
Others	55 (4.00)	40 (4.15)	15 (3.66)	
Size (cm)				0.476
0-3.9 cm	205 (14.92)	143 (14.83)	62 (15.12)	
4+ cm	380 (27.66)	258 (26.76)	122 (29.76)	
Unknown	789 (57.42)	563 (58.40)	226 (55.12)	
Laterality				0.983
One side	859 (62.52)	602 (62.45)	257 (62.68)	
Bilateral	515 (37.48)	362 (37.55)	153 (37.32)	
Surgery				0.314
No surgery/Biopsy	378 (27.51)	270 (28.01)	108 (26.34)	
STR	385 (28.02)	278 (28.84)	107 (26.10)	
GTR	611 (44.47)	416 (43.15)	195 (47.56)	

IQR, inter quartile range; STR, subtotal resection; GTR, gross total resection; NOS, not otherwise specified.

### LASSO Regression and Feature Selection

**Figure 2:**
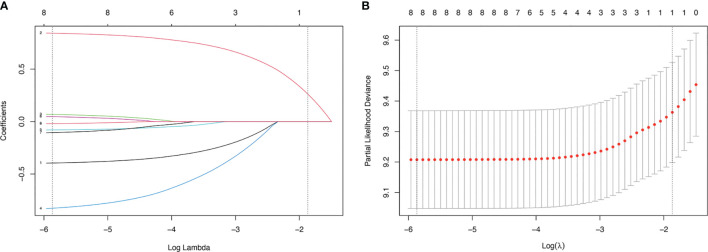
LASSO regression model was used to select characteristic impact factors. **(A)** LASSO coefficients of 8 features; **(B)** Selection of tuning parameter (λ) for LASSO model.

We used LASSO regression to screen for prognostic factors in WHO Grade III meningioma patients in the training cohort: gender (coefficient, -0.394), age (coefficient, 0.841), race (coefficient, 0.066), histology (coefficient, -0.822), location (coefficient, -0.078), size (coefficient, 0.045), laterality (coefficient, -0.103), surgery (coefficient, -0.019). The different colored lines in **Figure 2A** represent different variables: line 1 (black): gender, line 2 (red): age, line 3 (green): race; line 4 (blue): histology, line 5 (light blue): primary tumor site, line 6 (pink): tumor size, line 7 (black): tumor laterality, line 8 (red): surgical method. The lower horizontal axis in **Figures 2A, B** represents the magnitude of the lambda value in The LASSO regression model, and the upper horizontal axis represents the number of variables in the model whose coefficients are not zero at this time. The vertical dashed lines in **Figures 2A, B** represent the optimal value variables. We obtained 8 prognostic variables using the minimum standard value as the criterion.

### Construction of Prognostic Nomogram

**Figure 3:**
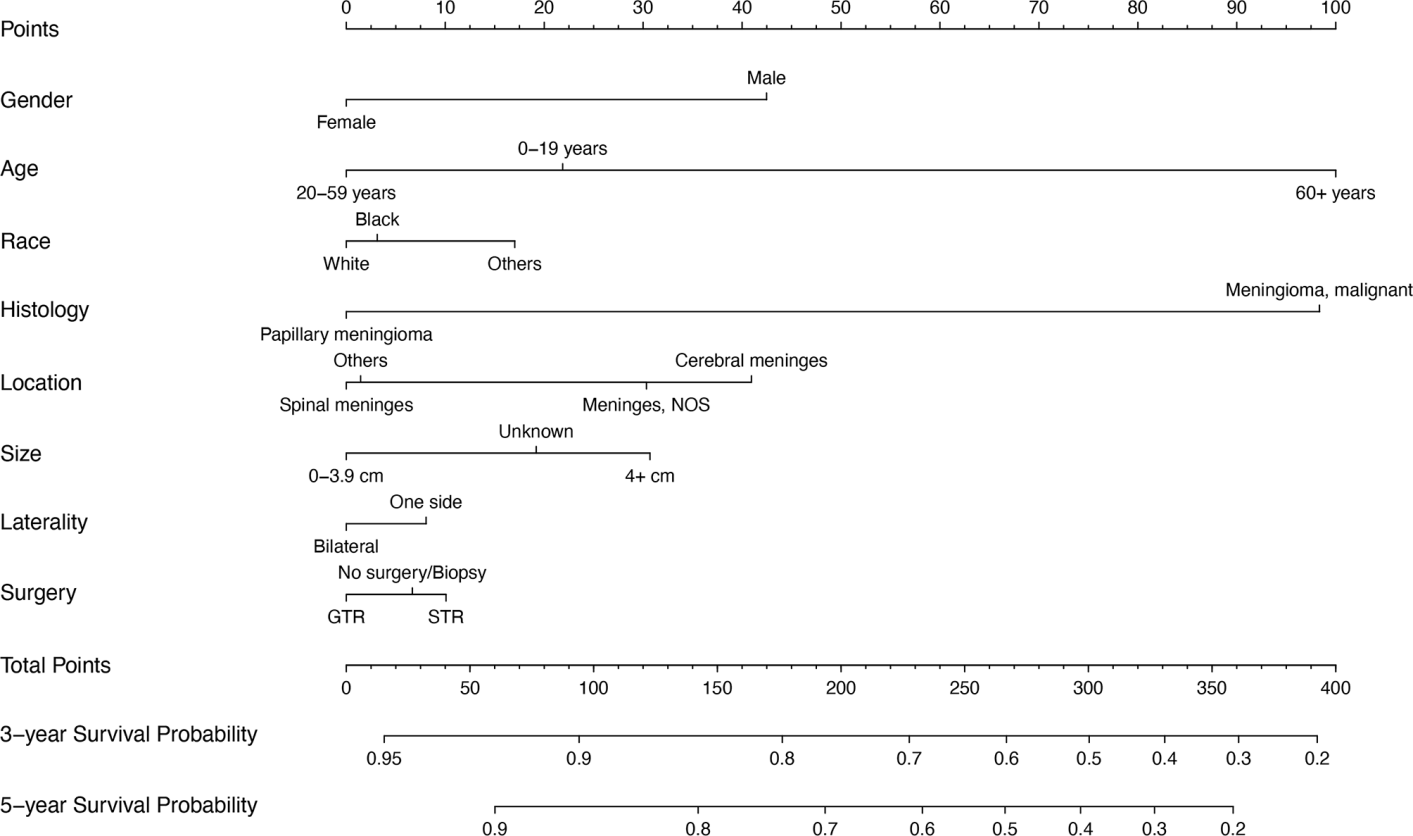
Prognostic nomogram of WHO Grade III patients with meningioma based on 8 risk factors. NOS, not otherwise specified; STR, subtotal resection; GTR, gross total resection.

The Cox regression model was constructed using the 8 variables selected. All variables passed the proportional hazards assumption test: gender (P=0.731), age (P=0.761), race (P=0.923), histology (P=0.082), location (P=0.448), size (P=0.106), laterality (P=0.468) and surgery (P=0.786). The 3- and 5-year survival probability of WHO Grade III meningioma was constructed using 8 variables (**Figure 3**). The detailed scores of each predictive variable in the histogram are presented in **Supplementary Table 1**.

### The Accuracy of the Nomogram

**Figure 4:**
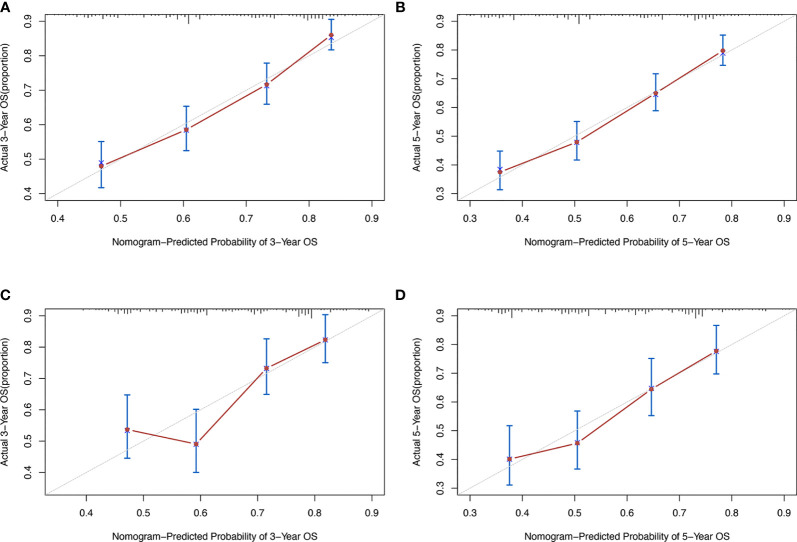
Calibration plot of the prognostic nomogram for each cohort. **(A)** 3-year calibration plot of the development set. **(B)** 5-year calibration plot of the development set; **(C)** 3-year calibration plot of the validation set; **(D)**. 5-year calibration plot of the validation set.

We calculated the C-index of the prognostic model of WHO Grade III meningioma in the development population and the validation population, which were 0.654 and 0.628, respectively. Development and validation cohort calibration plots at 3- and 5 years after diagnosis of WHO Grade III meningioma showed good agreement between prediction and actual observation (**Figure 4**).

### Decision Curve Analysis

**Figure 5:**
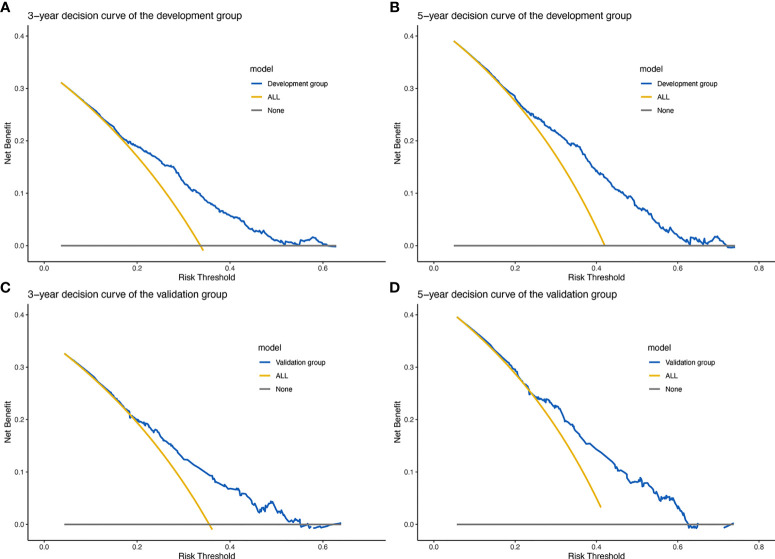
Prognostic decision curve analysis (DCA) of patients with WHO Grade III meningioma. **(A)** 3-year survival DCA of the development set; **(B)** 5-year survival DCA of the development set; **(C)** 3-year survival DCA of the validation set; **(D)** 5-year survival DCA of the validation set.

The clinical net benefit of the predictive model was assessed using DCA. The abscissa is the threshold probability and the ordinate is the net gain rate. If the dotted lines are higher than the two solid lines, the model is of clinical application value. The results show that the nomogram model has a good net benefit in predicting the 3- and 5-year survival of patients with WHO Grade III meningioma (**Figure 5**).

### ROC Curve of Survival Data

**Figure 6:**
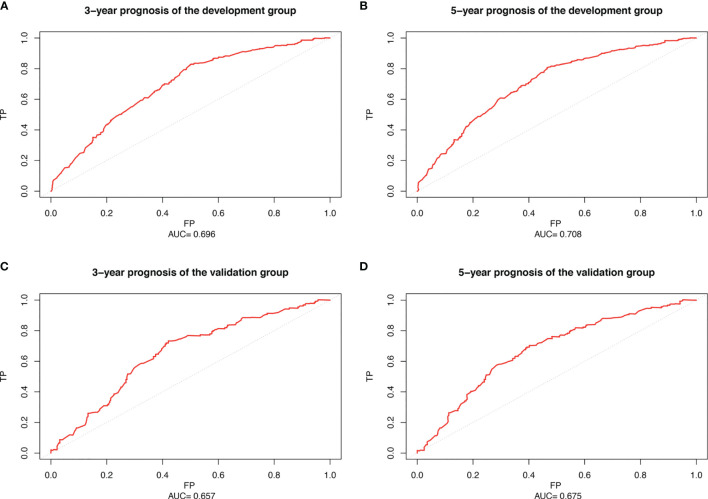
Receiver operating characteristic curve (ROC) of patients with WHO Grade III meningioma. **(A)** 3-year ROC of the development set; **(B)** 5-year ROC of the development set; **(C)** 3-year ROC of the validation set; **(D)** 5-year ROC of the validation set.

We used Cox proportional hazards regression model to predict the 3- and 5-year probability of death for each WHO Grade III meningioma patient. “survivalROC” package was used to draw the ROC of development and validation group at different time points with mortality as a continuous variable. AUC values were calculated. **Figures 6A, B** show the accuracy of the prognostic model in predicting 3- and 5-year mortality in the training set, with AUC values of 0.696 and 0.708, respectively. **Figures 6C, D** describe the validation set with AUC values of 0.657 and 0.675 at year 3- and 5-year. It indicates that the accuracy of the constructed model is moderately accurate (**Figure 6**).

### Survival Analysis

**Figure 7:**
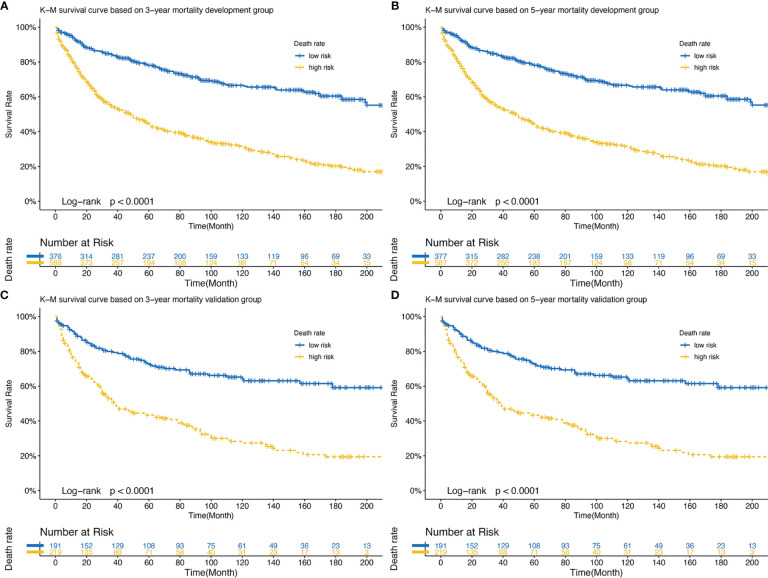
**(A)** Development set: K-M survival curve based on 3-year mortality; **(B)** Development set: K-M survival curve based on 5-year mortality; **(C)** Validation set: K-M survival curve based on 3-year mortality; **(D)** Validation set: K-M survival curve based on 5-year mortality. K-M, Kaplan-Meier.

Those whose mortality was greater than the cutoff point were defined as the high-risk group and the rest as the low-risk group and a log-rank test was used for comparison (**Figure 7**). The optimal cut-off points for 3- and 5-year mortality in the development cohort were 0.279 and 0.359. **Figure 7A** shows the development cohort probability of survival at 3-year in the low-risk group 0.84 (95%CI 0.81 to 0.88) and high-risk group 0.54 (95%CI 0.50 to 0.59). **Figure 7B** shows the development cohort probability of survival at 5-year in the low-risk group 0.78 (95%CI 0.74 to 0.83) and high-risk group 0.44 (95%CI 0.40 to 0.49). The optimal cut-off points for 3- and 5-year mortality in the validation group were 0.305 and 0.391. In the validation cohort, 3-year survival was 0.80 (95%CI 0.74 to 0.86) and 0.51 (95%CI 0.45 to 0.59) in the low-risk and high-risk groups, respectively (**Figure 7C**). 5-year survival was 0.73 (95%CI 0.66 to 0.80) and 0.43 (95%CI 0.37 to 0.51) in the low-risk and high-risk groups, respectively (**Figure 7D**).

## Discussion

This study is the first to develop and validate a clinical prognostic model for WHO Grade III meningioma based on the SEER database. To the best of our knowledge, this is the largest sample size study ever conducted. We constructed a visual nomogram to predict the 3- and 5-year survival probability of patients with WHO Grade III meningioma. As a graphical scoring tool for predictive models, the nomogram has been used to calculate survival probability and has become a part of modern medical treatment models (12). Through the evaluation of the model, it is verified that the nomogram has good accuracy and consistency, and can be used as an effective clinical prediction model in WHO Grade III meningioma.

We used a LASSO regression model to screen for eight variables with independent prognostic value for patients with WHO Grade III meningioma. Then the 3- and 5-year survival probability of WHO patients with meningioma were predicted by constructing a Cox proportional risk regression model. David et al. followed clinical data from 119 patients with atypical meningioma in a multicenter retrospective study (13). In univariate and multivariate analyses, they demonstrated that age greater than 60 years was an independent prognostic factor for patients with meningioma (13). Another retrospective study of 59 patients diagnosed with atypical or malignant intracranial meningiomas also concluded that patients younger than 40 years of age had a good prognosis (14). The prognostic nomogram of WHO Grade III meningioma patients constructed by us also confirmed that old age was an independent predictor of prognosis. Age as a general prognostic factor in cancer patients is also applicable in patients with meningioma. Many studies have illuminated the interaction between aging and the development of cancer, and mutations in oncogenes play a decisive role in the progression of cancer (15). Our study is consistent with previous findings that age plays a significant role in the prognosis of malignant meningioma.

In addition, histological classification is also a variable affecting the prognosis of patients with WHO Grade III meningioma. According to the WHO classification of central nervous system neoplasms published in 2016, three types of meningioma are classified as Grade III, including anaplastic meningioma (9530/3), papillary meningioma (9538/3), and rhabdoid meningioma (9538/3). Since the WHO classification is not completely consistent with the ICD-O-3, we classified WHO Grade III meningioma into two categories according to ICD-O-3 codes 9530/3 (Meningioma, malignant) and 9538/3 (Papillary meningioma). Nomogram indicated that the score of papillary meningioma (zero points) was significantly lower than malignant meningioma (98 points), indicating that patients with papillary meningioma had a better prognosis (**Supplementary Table 1**). Prospective studies of large papillary meningiomas are lacking and only a few case series have been reported (16, 17). As a rare variant subspecies of meningioma, its biological behavior still needs further exploration.

In a retrospective study of 44 patients with atypical meningioma, the extent of surgical resection was an important prognostic factor (18). Confirming that the surgical approach of total resection may lead to longer survival. In addition, a large cohort study of 302 patients with atypical meningioma also demonstrated a significant effect of total surgical resection on prognosis (19). Aizer et al. analyzed the clinical data of patients with atypical and malignant meningiomas from the SEER database and found that total resection of the tumor after adjustment for relevant confounding variables was an independent indicator of prognosis (P = 0.01) (20). Our study also confirmed that surgical treatment is an independent prognostic factor for WHO Grade III meningioma. The score of total resection group was lower than that of subtotal resection group, indicating that the prognosis of total resection group was better. The advantage of our study and previous studies is that the surgical procedures are presented with nomogram, which facilitates the calculation of 3- and 5-year survival rates.

In addition, this study showed that women had a better prognosis than men. A United States epidemiological survey of meningiomas also found a higher incidence of WHO II/III meningiomas in women aged 35-64 years than in men of the same age group (7). In the construction of another prognostic model for atypical meningioma, females also showed a benefit in overall survival. In general, gender is considered to have limited influence on the prognosis of patients with meningioma. However, the effect of gender on prognosis in WHO Grade III meningioma patients still needs further analysis.

According to the National Comprehensive Cancer Network (NCCN) guidelines, tumor size is recommended as an independent prognostic factor for tumors of the central nervous system (21). Our study shows that patients with malignant meningiomas larger than 4cm in diameter (31 points) have a poor prognosis. This is consistent with previous findings that larger tumors are also an adverse prognostic factor (20).

In fact, clinical application is an important criterion to verify the prediction performance of the nomogram. Moreover, we calculated that the nomogram had good accuracy in both the development and validation groups (C-index: 0.654 and 0.628). The ROC curves also confirmed the accuracy of the nomogram model prediction. DCA showed a good net clinical benefit from this nomogram (10). Overall, the nomogram of WHO Grade III meningioma has a good performance in practical application.

There are limitations to this study. First, we conducted a retrospective study using the SEER database, and the random allocation method may be biased. Further randomized controlled trials will be necessary to confirm the results. Second, meningiomas are usually classified according to WHO classification, which is not completely consistent with the ICD-O-3-based classification presented here. Third, the SEER database covers about 30% of the U.S. population, so the results limit generalization to other population groups. Finally, other important prognostic factors such as functional status, imaging, and humoral biomarkers were not available beyond the clinical data available in the registry.

## Conclusion

In summary, we developed and validated a nomogram for predicting 3- and 5-year survival in patients with WHO Grade III meningioma. This nomogram has sufficient predictive power and discrimination and good clinical application value. This nomogram may help clinicians provide personalized treatment and clinical decisions in patients with WHO Grade III meningioma.

## Data Availability Statement

The original contributions presented in the study are included in the article/**Supplementary Material**. Further inquiries can be directed to the corresponding author.

## Author Contributions

Study design: ZJ and YY. Methodological development: ZJ, YY, JW, HY, HZ, QC, and YH. Data acquisition and statistical analysis: ZJ and YY. Manuscript writing: All authors. Study Research guidance and supervision: YHH. All authors contributed to the article and approved the submitted version.

## Conflict of Interest

The authors declare that the research was conducted in the absence of any commercial or financial relationships that could be construed as a potential conflict of interest.

## Publisher’s Note

All claims expressed in this article are solely those of the authors and do not necessarily represent those of their affiliated organizations, or those of the publisher, the editors and the reviewers. Any product that may be evaluated in this article, or claim that may be made by its manufacturer, is not guaranteed or endorsed by the publisher.

## Supplementary Material

Additional File 1 Detailed scores for all variables in nomogram.

Additional File 2 Clinical data of WHO Grade III meningioma in development group.

Additional File 3 Clinical data of WHO Grade III meningioma in validation group.
