# Supplementary material for: Development and Validation of Prognostic Nomogram in Patients With WHO Grade III Meningioma: A Retrospective Cohort Study Based on SEER Database
Source: Front Oncol. 2021 Dec 1;11:719974. doi: 10.3389/fonc.2021.719974 (PMC8671456; doi:10.3389/fonc.2021.719974)
Supplement: Additional File 3 — Clinical data of WHO Grade III meningioma in validation group. [file Table_1.docx]

**Supplementary Table 1. Detailed scores for all variables in nomogram**

| **Variable** | **Nomogram Score** |
| --- | --- |
| Gender |  |
| Male | 42 |
| Female | 0 |
| Age |  |
| 0-19 years | 22 |
| 20-59 years | 0 |
| 65+ years | 100 |
| Race |  |
| White | 0 |
| Black | 3 |
| Others | 17 |
| Histologic |  |
| Meningioma, malignant | 98 |
| Papillary meningioma | 0 |
| Location |  |
| Cerebral meninges | 41 |
| Spinal meninges | 0 |
| Meninges, NOS | 30 |
| Others | 1 |
| Size (cm) |  |
| 0-3.9 cm | 0 |
| 4+ cm | 31 |
| Unknown | 19 |
| Laterality |  |
| One side | 8 |
| Bilateral | 0 |
| Surgery |  |
| No surgery/biopsy | 7 |
| STR | 10 |
| GTR | 0 |

NOS: not otherwise specified; STR: subtotal resection; GTR: gross total resection
